# Not Everything Requires a Psychiatry Consult: Subdural Hematoma as a Cause of Transient Acute Quadriplegia

**DOI:** 10.7759/cureus.12104

**Published:** 2020-12-15

**Authors:** Ryan F Amidon, Christ Ordookhanian, Paul Kaloostian

**Affiliations:** 1 Neuroscience, University of California Riverside, Riverside, USA; 2 Medicine, University of California Riverside School of Medicine, Riverside, USA; 3 Neurological Surgery, Riverside Community Hospital, Riverside, USA; 4 Neurological Surgery, Paul Kaloostian M.D. Inc., Riverside, USA

**Keywords:** subdural hematoma, altered mental status, quadriplegia, craniotomy, hematoma evacuation

## Abstract

While subdural hematoma (SDH) is a commonly encountered emergent pathology that is often in the setting of trauma, its ability to present with a wide variety of symptoms, or no symptoms at all, may delay diagnosis. SDH symptoms progress in a stepwise manner, potentially resulting in rapid neurological degeneration and can result in irreversible damage. Here we describe a case of an elderly woman with bilateral chronic SDH with mass effect who initially presented with severe headaches and a mild altered mental status, notably without a history of head trauma. Diagnosis was achieved through radiographic imaging. Within 24 hours, the patient suddenly became quadriplegic. Emergent bilateral evacuation of SDH was performed. Full neurological recovery of both arms and legs was achieved without delay, demonstrating the ability of this approach to reverse the development of acute quadriplegia attributed to SDH in such patients. Comprehensive and timely medical screening on initial presentation accompanied by radiographic studies, especially of patients presenting with altered mental status is crucial for identifying any underlying pathology, such as SDH. Altered mental status without head trauma is not always psychologic in nature and a psychiatric consult is insufficient in identifying lesions of the central nervous system (CNS). Altered mental status encompasses a broad differential diagnosis that seeks to find organic causes of altered state. While mortality from symptomatic chronic SDH is high, especially in the geriatric patient population, our findings support the position that rapid diagnosis and intervention to reverse neurological deterioration is an essential component of improving patient outcomes.

## Introduction

Subdural hematoma (SDH) is a relatively common pathology encountered in the acute emergency setting that may be asymptomatic or symptomatic, with a vast array of symptoms possible. The risk of acquiring SDH increases significantly with age; however, it may still occur in younger age brackets. Reported incidence rates range from 1.72 to 20.6 cases per 100,000 persons each year [1]. Hsieh et al. report a much higher incidence of 8 to 58 per 100,000 in patients older than 65 years old, considered elderly, compared to an incidence of 3.4 per 100,000 in younger patients [2]. The incidence of SDH has been steadily increasing over time in many countries, which can be attributed to aging populations and the growing use of anticoagulation and antiplatelet medications [1,3]. According to the United States Census Bureau, the elderly population grew by 34% between 2010 and 2019 [4]. In 2016, the elderly comprised just over 15% of the total United States population and it is projected that by 2030, they will constitute over 20% of the population; by 2035, the elderly population will supersede the population under 18 years old [5]. In light of these projections, it is likely that the frequency of SDH cases will continue to grow.

Unfortunately, the prognosis from symptomatic SDH is poor with mortality rates as high as 40% to 60% [6]. There is a marked difference between age brackets, however: mortality was 75% in patients over 50 years old compared to 25% in patients between 10 and 30 years old with chronic SDH. Considering the high mortality, especially in the elderly, and the impending aging population, appropriate management of the many possible manifestations of this pathology is very important. This case highlights the successful management of an elderly patient with bilateral chronic SDH who presented with severe headaches and a mild altered mental status, subsequently succumbing to transient acute quadriplegia, requiring emergent neurosurgical intervention.

## Case presentation

**Figure 1:**
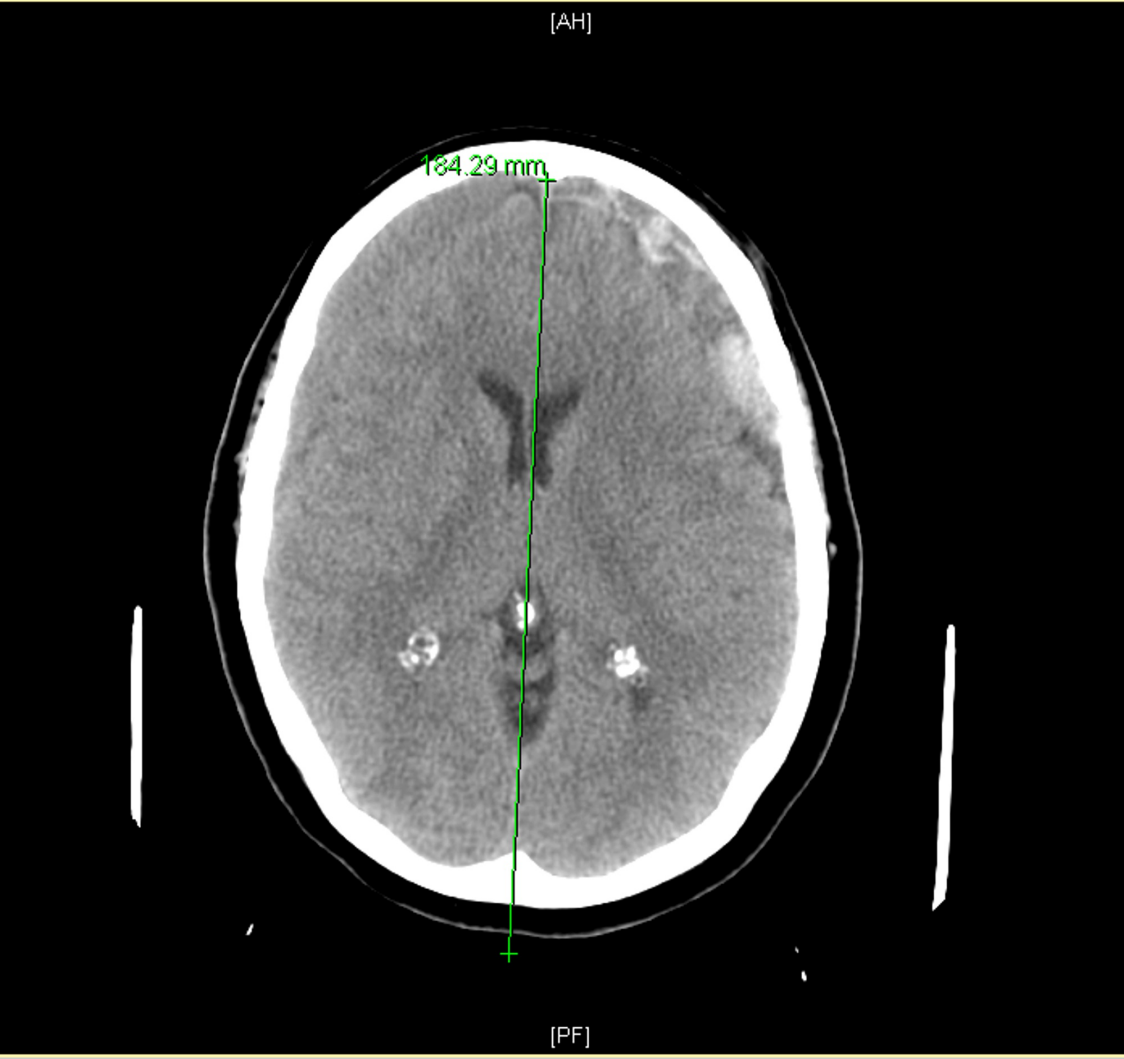
Preoperative head CT scan reveals bilateral SDH with mass effect. Effacement of the bilateral sulcal gyral pattern is observed CT: Computed tomography; SDH: Subdural hematoma.

A 65-year-old female patient presented to the emergency department with severe headaches and a mild altered mental status, without a history of head trauma. There were no focal neurological deficits upon examination. Her vitals, complete blood count (CBC), comprehensive metabolic panel (CMP), magnesium, phosphate, liver function tests (LFTs), coagulation studies, lipase, troponin, thyroid function, urinalysis (UA), and urine toxicology were normal. She was not using any anticoagulation medication and she was ambulatory. A computerized tomography (CT) scan of the head revealed bilateral SDH with mass effect bilaterally causing effacement of the bilateral sulcal gyral pattern (Figure 1).

**Figure 2:**
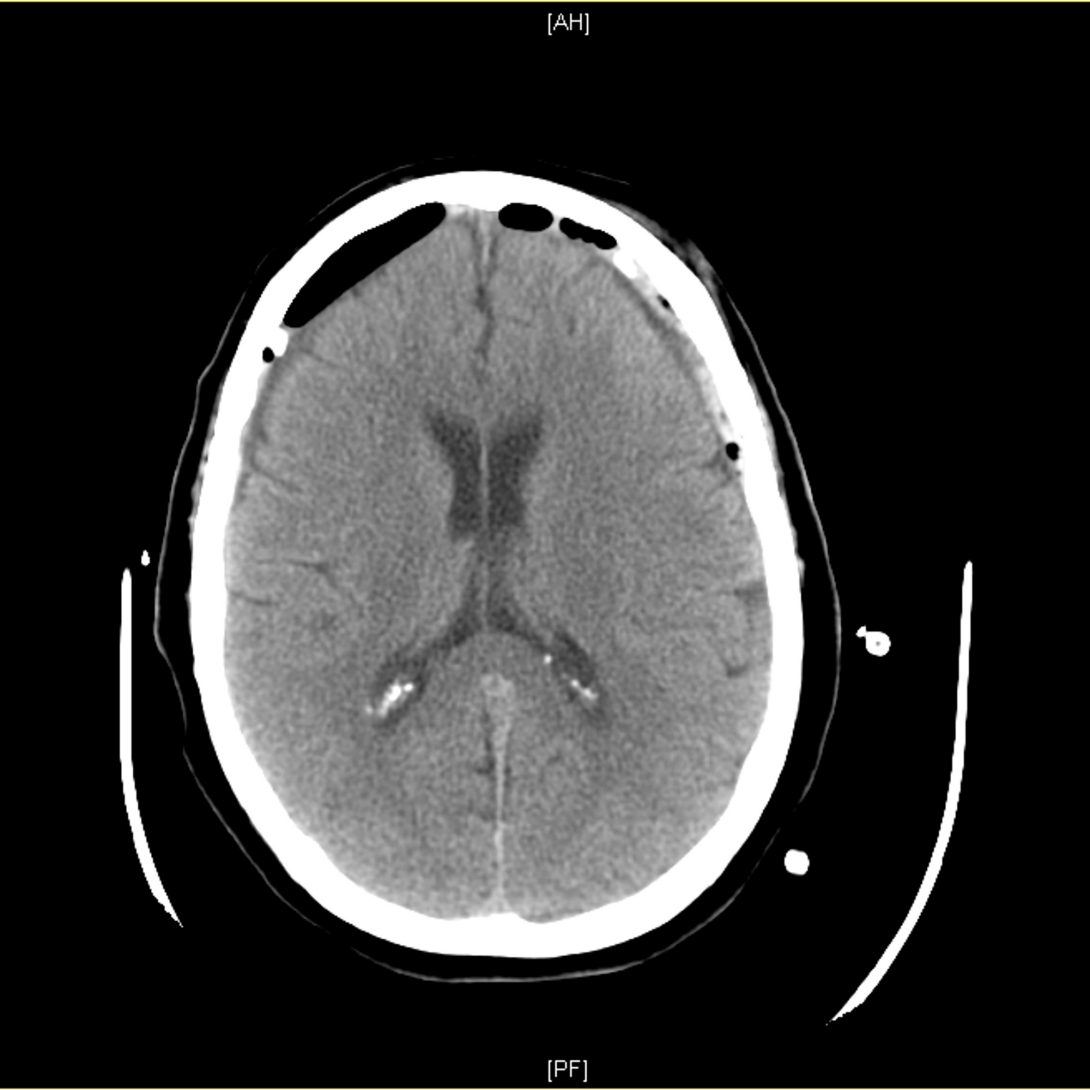
Postoperative head computed tomography (CT) scan reveals successful decompression of the brain bilaterally

The patient was placed on levetiracetam for seizure prophylaxis. She was recommended surgery, but decided against it and requested to be discharged home. A few hours later, prior to leaving the hospital, the patient became acutely quadriplegic. She was noted to have 1/5 strength in both arms and legs and was speaking appropriately with normal mental status. An urgent head CT scan was ordered, revealing unchanged bilateral SDH. The patient then was counseled and recommended for an emergent surgery, which included emergency bilateral craniotomies and SDH evacuation. A postoperative CT scan of the head demonstrated appropriate decompression of the brain bilaterally (Figure 2).

The patient immediately returned to her baseline without any neurological deficits.

## Discussion

A hematoma develops when blood collects outside of the blood vessels. The brain is covered by three meningeal layers: the dura, the arachnoid mater, and the pia mater, the dura being closest to the inner skull. An SDH refers to a collection of blood between the dura and the arachnoid mater. The most common etiology is head trauma, which constitutes 50% to 80% of cases [1-2]. SDH is a complication in 11% to 20% of head and brain injuries, depending on their severity. Other potential causes include antithrombotic medications (including aspirin and anticoagulants), cerebral atrophy, intracerebral hemorrhage, ruptured cerebral aneurysm, brain tumor, cerebral vascular malformations, vasculopathy, coagulopathy, systemic thrombolysis, and intracranial hypotension [1]. Rarely, an SDH may also develop as a complication of craniotomy in patients with unruptured cerebral aneurysms [7]. Due to the vast array of potential causes, the pathogenesis of SDH has been studied for decades and numerous theories have been proposed.

The advent of CT technology has catalyzed SDH research, significantly advancing our understanding of the pathology. Tanaka and Ohno explained that tears in the arachnoid membrane initiate SDH development, resulting in bloody cerebrospinal fluid (CSF) collecting into subdural space [7]. As this fluid collects over time, an inflammation reaction occurs, creating a neomembrane at the inner surface of the dura. Inflammatory cytokines in the fluid may induce this effect. Inside of the neomembrane, recurrent bleeding from sinusoid channels occurs as a result of a cycle of local angiogenesis, inflammation, coagulation, and fibrinolysis [3]. New blood vessels form, inflammation and coagulation ensue, producing fibrin to promote blood clotting, and fibrinolysis breaks down the fibrin, countering the clotting process [8]. Fibroblasts spread over the inner dural surface, supporting the formation of a thick outer membrane and a thin inner membrane, encapsulating the hematoma [1]. The outer membrane harbors cytokines that trigger cyclooxygenase-2 (COX-2) to synthesize prostaglandin E2 (PGE2), inducing inflammation [7]. The growing density of capillary vasculature in the capsule from local angiogenesis sets the stage for repetitive bleeding events; in conjunction with inflammation, the hematoma enlarges, potentially becoming symptomatic or increasing severity of symptoms [9]. In some cases, spontaneous resolution may occur when the sources of bleeding are naturally eliminated [7]. 

Potential symptoms from SDH are even more numerous than potential causes. They include confusion, headaches, cognitive impairment, apathy, seizure, aphasia, homonymous hemianopia, dementia, hemiparesis or hemiplegia, and quadriparesis or quadriplegia [10-11]. Symptoms may only begin to develop weeks or months after the initial causal incident (i.e., trauma). Quadriparesis and quadriplegia are rather unusual manifestations of chronic SDH, whereas headaches and confusion are relatively common. The precise mechanisms that result in limb weakness and loss of function are unclear. Lesoin et al. provided several theories: arm deficits may be a result of direct compression of cerebral hemispheres; however, the cortical regions responsible for lower limb movement are relatively protected from such compression [11]. Leg deficits may instead be a result of ischemic events, brainstem compression, or compression of veins draining from the Rolandic zone to the superior longitudinal sinus. The first theory would involve ischemia of the frontal lobe, which should simultaneously disturb one’s state of consciousness. The second theory should result in other brainstem symptoms accompanying the leg weakness. The authors believe that the specific mechanisms involved may change on a case-by-case basis. Lesoin et al. postulated that the stepwise course of symptoms, as evidenced by sudden neurological decline, may be a consequence of recurrent bleeding events within the hematoma capsule [11]. This is supported by the finding that within capsules of chronic SDH, when neurological decline is present, the capillary walls demonstrated high permeability as capillary endothelial cells degenerated [9].

Diagnosis of SDH can be accomplished through a variety of modalities. Radiological approaches include head CT without contrast, magnetic resonance imaging of the head without contrast, and a head X-ray to test for skull fracture if indicated by a history of traumatic injury. Other approaches include physical examination, neurological examination, and the Glasgow Coma Scale (GCS) to measure the state of the patient’s consciousness. The GCS is conventionally used in patients who experienced head trauma.

Head trauma, or traumatic brain injury (TBI), is not only the most common cause of SDH but its most obvious outcome is altered mental status [12]. TBI can be described as impact (the head makes contact with another object) or non-impact (the head receives a non-impact force such as quick acceleration and deceleration) [13]. Symptoms characteristic of mild to moderate TBI include headaches, dizziness, nausea, fatigue, blurred vision, sleep disturbances, and amnesia [13-15]. The mechanisms that ultimately result in altered mental status are complex, involving numerous pathological cellular pathways initiated by disruptions in ion and neurotransmitter concentrations. Prins et al. explained that cell membrane potentials are altered and glutamate is released. Extracellular potassium and intracellular calcium concentrations increase. The latter overloads the mitochondria, inducing oxidative stress and disrupting mitochondrial function. Additionally, acute cerebral hyperglycolysis occurs, which may impair cerebral blood flow [13].

Over 10 million annual TBI cases occur internationally, with one case transpiring every 15 seconds in the United States [12-13]. Similarly, patients presenting with altered mental status are relatively common in the emergency department. Korn et al. described it as the most common initial complaint requiring medical evaluation at the Los Angeles County and University of Southern California Medical Center [14]. Altered mental status is useful in diagnosing TBI and determining severity, which is quantified using the GCS, which ranges from 3 to 15. The score is determined by the patient’s verbal response, eye opening, and motor activity [12]. A score of 3 to 8 is severe, 9 to 12 moderate, and 13 to 15 mild [13]. In the absence of head trauma, however, the etiology of altered mental status is not always so clear.

Altered mental status is a very broad term that includes, but is not limited to, confusion, strange behavior, lethargy, agitation, psychosis, disorientation, and hallucination [15]. These are particularly alarming when they are acute manifestations as they are considered more likely to represent life-threatening conditions [16]. The etiology of altered mental status is not always psychiatric in nature, meaning a psychiatry consult is infrequently sufficient and may not even be required [15]. Upon presentation to the emergency department, the patient should receive a thorough evaluation incorporating a history, a physical exam, and laboratory and radiographic testing [16]. Fortunately, the Consolidated Omnibus Budget Reconciliation Act (COBRA) and the Emergency Medical Treatment and Active Labor Act (EMTALA) require appropriate screening for all patients seeking treatment in the emergency department [14]. For patients with altered mental status without a clear etiology, this should include radiographic testing.

Numerous treatment strategies exist for chronic SDH, including burr-hole evacuation for uncomplicated cases, craniotomy for symptomatic patients, medical management to reduce secondary damage (i.e., antiseizure medication) in patients deemed poor candidates for surgery, and general nonsurgical management for asymptomatic cases [17]. Surgical intervention is recommended when moderate to severe cognitive impairment, progressive neurological deterioration, or midline shift of 5 mm or higher accompanies the SDH [6]. Recurrence of chronic SDH is reported in 5% to 30% of patients, with rates highest in patients with elderly status, thick hematoma width, bilateral presentation of SDH, and use of antiplatelet and anticoagulant medication [6]. Postoperative drainage is linked with a reduced risk of recurrence.

Bilateral SDH is an uncommon presentation, accounting for merely 14% to 25% of chronic SDH cases [2]. Symptoms of nausea, vomiting, headache, and unsteady gait are more common with this manifestation of SDH. Unfortunately, bilateral SDH is associated with significantly worse outcomes than unilateral SDH [18]. While the frequency of focal neurological impairments is lower than with unilateral SDH, this may confound diagnosis and delay treatment [19]. Additionally, the negative effects of brain herniation are more pronounced with bilateral SDH and there is a higher recurrence rate postoperatively [18-19].

Our patient’s advanced age represented a risk factor for chronic SDH. After presenting with severe headaches and an altered mental status, comprehensive medical screening was conducted, which included a CT scan of the head to analyze the potential presence of one or more brain lesions. If this was foregone under the assumption that the patient’s mental state was a result of a psychiatric condition in the absence of head trauma, the diagnosis of bilateral SDH would have been severely delayed. Since the patient initially declined surgery, we administered Keppra (levetiracetam) as a preventative measure against seizure development; however, the abrupt onset of acute quadriplegia indicated surgical intervention. The validity of this approach is supported by the literature demonstrating significantly improved neurological status in patients with quadriparesis and quadriplegia after hematoma evacuation [11,20]. Our patient fully regained neurological function of her arms and legs. Our success provides useful lessons for not only treating patients with acute quadriplegia from bilateral SDH but also the importance of testing for brain lesions in patients with altered mental status without head trauma.

## Conclusions

SDH is a relatively common pathology encountered in neurosurgery, especially in elderly patients. As the proportion of the geriatric population is increasing with time in many countries, SDH will likely become more prevalent. This case demonstrates that when a patient presents with an altered mental status and there is no history of head trauma, radiographic testing is crucial to identify underlying conditions such as SDH. A psychiatric consult alone is insufficient and often not required. Additionally, our intervention demonstrated that an acute onset of quadriplegia associated with bilateral SDH can be reversed through immediate neurosurgical intervention. Emergent bilateral craniotomy and hematoma evacuation set the stage for immediate postoperative recovery despite this unusual clinical manifestation frequently resulting in poor outcomes. We believe that our case will provide the insight necessary to diagnose and treat patients with bilateral chronic SDH.
